# Bacterial Detection and Recovery From Poultry Litter

**DOI:** 10.3389/fmicb.2021.803150

**Published:** 2022-01-06

**Authors:** Jodie R. Plumblee Lawrence, Denice Cudnik, Adelumola Oladeinde

**Affiliations:** U.S. National Poultry Research Center, USDA-ARS, Athens, GA, United States

**Keywords:** poultry litter, food-borne pathogens, limit of detection, limit of quantitation, food safety, method validation

## Abstract

The level of pathogens in poultry litter used for raising broiler chickens is critical to the overall health of a broiler chicken flock and food safety. Therefore, it is imperative that methods used for determining bacterial concentration in litter are accurate and reproducible across studies. In this perspective, we discuss the shortcomings associated with current methods used for bacterial quantification and detection from litter and assess the efficacy of one method for pathogen and commensal (*Campylobacter*, *Salmonella*, *Escherichia coli*, and *Enterococcus* spp.) recovery. The limit of quantitation and detection for this method differed between pathogens, and the recovery rate (∼138–208%) was higher for *Salmonella*, *E. coli*, and *Enterococcus* compared to *Campylobacter* (24%). Our results suggest that pathogen recovery from litter is highly variable and pathogen concentrations need to be reported in dry weight before comparisons can be made between studies.

## Introduction

Poultry litter is a complex material comprised of decomposing plant-based bedding (e.g., wood shavings, sawdust, and rice or peanut hulls) mixed with chicken feces, uric acid, feathers, feed, insects, and other broiler-sourced components. Therefore, poultry litter carries a unique and complex population of bacteria, fungi, and viruses (Martin et al., 1998; Terzich et al., 2000; Dumas et al., 2011; Wadud et al., 2012). Since it is commonly used for raising broiler chickens, poultry litter is the first non-self-biological active substance that is ingested by a chick after it is introduced to a grow-out farm. Consequently, bacteria present in litter are the first inocula that will colonize the gut of broiler chicks; therefore, it is important to accurately determine if food-borne pathogens are present in litter before, during, and after use. Moreover, the continued withdrawal of antibiotics from preharvest poultry production has increased the focus on “proper” litter management for pathogen and disease reduction. However, detecting or enumerating bacterial pathogens in such a complex and heterogeneous mixture as litter can be challenging. Consequently, various methods have been used to isolate pathogens such as *Campylobacter* and *Salmonella* (Read et al., 1994; Kiess et al., 2010; Brooks et al., 2016) and commensals like *Escherichia coli* and *Enterococcus* (Terzich et al., 2000; Khan et al., 2005; Graham et al., 2009; Chinivasagam et al., 2016). This lack of a gold standard method can lead to invalid conclusions on pathogen presence and make published results incomparable across studies and samples.

To provide a narrative perspective to this problem, we surveyed published studies that reported data on *Salmonella* present in poultry litter and determined the methods used for *Salmonella* detection and recovery. To compare the methods, we used the minimum number of organisms in a sample the method could accurately quantify, i.e., limit of quantitation (LOQ), and the minimum number of organisms that can be detected in a sample using a given method, i.e., limit of detection (LOD). Furthermore, we performed an *in vitro* inoculation of oven-dried litter to shed light on the inherent variability of bacteria recovered from litter. By showing the variability both in the literature and from a spiked poultry litter experiment, this perspective aims to promote a discussion on this problem and encourage additional collaborative research to address the complexities of the issue.

## Materials and Methods

### Survey of Methods Used for *Salmonella* Isolation From Broiler Litter

Using keywords “Salmonella + broiler litter” or “Salmonella + poultry litter,” we searched PubMed, Scopus, and GoogleScholar for studies published between 2010 and 2020 that reported methods used for isolating *Salmonella*. We selected 27 studies (Supplementary Table 1) that noted how litter was characterized including the media used for bacterial isolation and enumeration and the LOQ and LOD of the method, and how *Salmonella* abundance was normalized, i.e., if *Salmonella* concentration was corrected for moisture levels. We chose *Salmonella* because it is one of the primary food-borne pathogens that are commonly linked to poultry.

### Poultry Litter Inoculation Study

We inoculated oven-dried litter with relevant, poultry-associated bacterial species. Reused litter (composed of decomposed pine shavings) was collected after broiler chicks were removed and stored at 4°C for several weeks before it was used for this study. To determine litter pH, litter eluate was made by adding 20 ml of 1X PBS to a whirl pak bag containing 5 g of litter and hand massaged for 1 min before shaking on a platform rocker for 14 min. The pH of the litter eluate was determined with an Orion Star Portable pH Meter (Thermo Fisher Scientific, Waltham, MA, United States). Litter moisture was determined gravimetrically as described previously (Oladeinde et al., 2018a). Reused litter was dried in an oven (moisture content was ∼10.1% and pH was ∼7.8 before drying) for 48 h at ∼105°C to effectively kill any indigenous bacteria. To ensure no pathogens were present in the dried litter, we performed bacteriological analysis on litter aliquots as described in the culture methods below.

A cocktail consisting of 16 bacterial strains (Supplementary Table 2) isolated from poultry litter or chicken carcass rinses was freshly prepared. All strains except *Campylobacter* were cultivated at 37°C for 18 h at ∼140 rpm in an orbital shaker. Colonies were taken from tryptic soy agar (TSA) with sheep blood and separately inoculated into 5 ml Luria Bertani (LB) Broth in a 15 ml conical tube. After incubation and shaking, cells were pelleted, washed with phosphate-buffered saline (PBS), pelleted again, and resuspended in PBS. For *Campylobacter*, freshly grown colonies from a Cefex plate (Remel, Lenexa, KS, United States) were used to generate a suspension in Mueller Hinton (MH) broth. Equal volumes of each strain were combined to produce a cocktail with each organism at ∼10^8^ cfu/ml. Serial dilutions of the cocktail (∼10^7^ to 10^1^ CFU/ml) were prepared in 1X PBS (Supplementary Figure 1). Each dilution (20 ml) was used to inoculate litter (5 g) in triplicate resulting in concentrations ranging from 10^6^ to 1–6 CFU/strain per g of litter. Serial dilutions were also plated in duplicate on selective agar to verify inoculum amounts. Spiked litter was hand massaged for 1 min and then placed on a platform rocker (∼40 rpm) for 14 min (Supplementary Figure 1).

Litter slurry aliquots (100 μl) were serially diluted, direct plated, and enumerated on selective agar: Cefex for *Campylobacter*, CHROMagar ECC (DRG International, Springfield, NJ, United States) for *E. coli*, m-Enterococcus (Neogen, Lansing, MI, United States) for *Enterococcus*, and BG Sulfa (Becton Dickinson, Franklin Lakes, NJ, United States) for *Salmonella*. Cefex plates were incubated at 42°C under microaerobic (5% O_2_, 10% CO_2_, and 85% N_2_) conditions for 48 h. All other plates were incubated at 37°C for 24–48 h before enumeration. Blue-green colonies on CHROMagar ECC were considered *E. coli*, pink to maroon colonies on m-Enterococcus agar were considered *Enterococcus*, and light pink colonies were considered *Salmonella* on Brilliant Green Sulfa (BGS). Pink or grayish colonies on Cefex were considered *Campylobacter*. Dilutions with at least two out of three replicates containing two or more colonies per plate were included in LOQ calculations (Supplementary Table 3).

Additionally, enrichment broth was plated onto appropriate selective agar for any sample negative by direct plating. For *Campylobacter*, spiked litter was diluted 1:10 in Bolton broth [*Campylobacter* enrichment broth (Neogen, Lansing, MI, United States), 5% lysed horse blood and Bolton broth selective supplement (Oxoid)] and incubated at 42°C under microaerobic conditions for 48 h. For the other bacteria, spiked litter was diluted 1:10 in buffered peptone water (BPW) and incubated at 37°C for 18–24 h. After transfer of enriched culture, selective agar plates were incubated as above and noted for the presence/absence of growth. The lowest inoculum amount with at least two out of three replicates positive for bacterial growth after enrichment and plating was considered the detection limit.

The LOQ was determined for each organism as the lowest inoculum amount that gave countable plates (2–300 colonies) for two out of three replicates after direct plating (Supplementary Table 3). The LOD was determined as the lowest inoculum amount that produced growth in at least two out of three replicates after culture enrichment.


$$\mathrm{Percent}\,\mathrm{recovery}\,\left(\mathrm{mean}\,\mathrm{of}\,\mathrm{three}\,\mathrm{replicates}\,\mathrm{for}\,\mathrm{each}\,\mathrm{inoculum}\,\text{amount}\right)=\frac{\mathrm{concentration}\,\mathrm{of}\,\mathrm{bacteria}\,\mathrm{recovered}\,\mathrm{from}\,\mathrm{inoculated}\,\mathrm{litter}\,\left(\text{cfu}/\text{g}\right)}{\mathrm{concentration}\,\mathrm{of}\,\mathrm{inoculum}\,\mathrm{added}\,\mathrm{to}\,\mathrm{the}\,\mathrm{litter}\,\left(\text{cfu}/\text{g}\right)}$$

Continuous variables did not meet the assumption of a normal distribution; therefore, non-parametric testing for direct comparisons was used for one-way analysis of variance tests. To determine if there was a significant difference in recovery between bacterial genera, and if the inoculum size significantly affected bacterial recovery, we performed a Kruskal–Wallis rank-sum test on log-transformed percentage of inoculum recovered. Statistical tests were performed using R (version 3.4.1).

## Results and Discussion

Proper litter management during pre-harvest is critical to the overall well-being of each broiler flock and for food safety. Broiler chickens that were positive for *Salmonella* at pre-harvest are more likely to carry *Salmonella* after processing (Volkova et al., 2010, 2011). However, our research on understanding how and what makes pathogens such as *Salmonella* and *Campylobacter* persist in broiler houses remains elusive. Multiple factors can be attributed to the lack of research in this field including the complexities of the poultry industry and the “heterogeneity” of poultry litter. Poultry litter is an important experimental unit for monitoring the prevalence and emergence of pathogens before, during and after grow-out periods. As there is no standard methodology, manipulating litter for pathogen detection represents one of the major constraints of litter research. Consequently, studies that have investigated pathogen concentration in litter vary considerably in the methods used.

We searched the literature for methods that were used for recovering *Salmonella* from poultry litter. We found 27 studies that measured *Salmonella* levels in litter: 14 studies were from litter composed of wood or pine shavings, 1 from rice hull and 12 did not report the litter type. For litter processing and enrichment, 17 different types of media were reported with the most common being BPW (*n* = 13 studies) (Supplementary Table 1). A large assortment of media was used for enumeration, with no clear explanation for choosing one selective medium over another. Most enumeration methods, either direct plating or some form of most probable number (MPN), involved enrichment in BPW, followed by selective enrichment in Rappaport-Vassiliadis broth (RV) and/or Tetrathionate Broth (TT) before plating onto Brilliant Green Agar (BGA)/BGS and/or Xylose Lysine Deoxycholate (XLD)/Xylose Lysine Tergitol 4 (XLT-4) agars. Some methods required that the litter be stomached, shook, or mixed after the enrichment broth was added, but the duration reported varied between studies (Supplementary Table 1). Nine studies reported a LOD or LOQ for their method (range; <1–100 CFU or MPN/gram). However, over half (*n* = 18) of the studies did not report a LOD or LOQ and no study reported the recovery efficiency of the method used. Together, these observations revealed no adequate metrics exist to ascertain if one method performs better than another.

**FIGURE 1:**
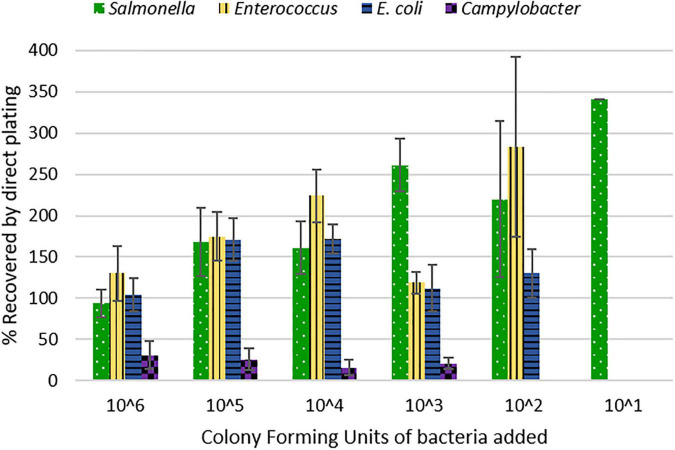
Percent of bacterial inocula recovered from litter.

Therefore, to start a discussion on the complexities of bacterial recovery from litter and encourage further research, we conducted a spiked poultry litter experiment. We inoculated oven-dried litter with a consortium of relevant poultry-associated bacterial species at six different concentrations (Supplementary Figure 1). We assumed that the oven-dried litter used was 100% dry weight and that the microbiota present comprised solely of inoculated bacterial strains. The recovery efficiency of the method examined differed between bacteria (X_2_ = 31.013, df = 3, *p*-value = 8.488e-07) (Table 1). *Salmonella* exhibited the highest percent recovery, followed by *Enterococcus*, *E. coli*, and *Campylobacter*. The method recovery efficiency also differed by the concentration of inoculum added (X_2_ = 15.07, df = 5, *p*-value = 0.01). For instance, we recovered >200% [coefficient of variation (CV) = 43%] of *Salmonella* cells inoculated when ∼ 10^2^ CFU/g litter was used as inoculum size, compared to 94% (CV = 18%) recovery when 10^6^ CFU/g litter was used. This inoculum-based difference was also true for *Enterococcus* and *E. coli* (Figure 1). For *Campylobacter*, we could only recover a maximum of around 31 ± 17% of the inoculum added. Furthermore, the highest LOQ (1600 cfu/g) and LOD (160 cfu/g) was for *Campylobacter*. *Salmonella* had the lowest LOQ (44 cfu/g), while *Enterococcus* had the lowest LOD (∼2 cfu/g) (Table 1 and Figure 1).

**TABLE 1:** Limit of quantitation, detection, and percent recovery for this method.

	LOQ (cfu/g litter)	LOD (cfu/g litter)	Average % Recovery (± SD)
*Campylobacter*	1600	160	24 ± 12^a^
*E. coli*	680	6.8	138 ± 36^b^
*Enterococcus*	230	2.3	186 ± 78^b^
*Salmonella*	44	4.4	208 ± 86^b^

*LOQ, limit of quantitation; LOD, limit of detection; SD, standard deviation.*

*Average values within columns with no like superscripts are significantly different (p ≤ 0.05).*

Although it is not theoretically possible to recover >100% of the inoculum added to litter, our data showed that this was the case. One plausible explanation is that bacterial growth occurred throughout the process of preparing the inoculum, making dilutions, mixing with litter, and spread plating on agar. Even though the process took less than an hour, bacteria have been shown to substantially increase in concentration within short periods of time (Oladeinde et al., 2018b; Chase et al., 2019). Alternatively, the large volume of PBS (20 ml) used for spiking the litter (5 g) could create an artificially high microbial load in the saline, as the bacteria would have been more tightly associated with the litter in a naturally occurring environment.

The dearth of broiler litter studies reporting measures for method validation makes it impossible for us to compare this method to other published methods. Previous studies (Supplementary Table 1) that determined *Salmonella* concentration in litter grab samples reported the LOQ and LOD of those methods to be between 0.3 and 100 CFU or MPN per gram of litter. Our method revealed that there is high variability and difficulty associated with detecting and quantifying pathogens present in poultry litter. The method’s variability was dependent on inoculum concentration and *Salmonella* had the highest variability (Figure 1), which was also the bacteria with the highest recovery. Contrastingly, *Campylobacter* results were less variable but had the lowest recovery in litter. Studies have reported that *Campylobacter* might not be readily detected in litter with culture methods, so DNA-based techniques such as PCR have been recommended (Brooks et al., 2015; Kassem et al., 2017). Another source of variability could be the different media we used for inocula preparation. Cefex agar and MH broth were used for *Campylobacter* strains, while TSA and LB broth were used for other bacterial strains.

Furthermore, *Salmonella* concentration was not normalized by the litter moisture content in the majority of studies reviewed. Moisture content of litter can range from 10 to 70% depending on a variety of factors: sample location within broiler house, number of flocks raised on litter, and if/how composted (Martin et al., 1998; Furtula et al., 2010; Miles et al., 2011; Pepper and Dunlop, 2021). *Salmonella* numbers reported on a dry weight basis are higher than the same result when reported as wet weight or “as-is” basis. The uncorrected bacteria concentration is biased low because it includes bacteria in the moisture or other liquid phase of the litter. The degree of bias in the pathogen concentration reported is affected by the moisture content of the litter sample. For example, a litter that has 75% moisture and was reported to carry 30 CFU of *Salmonella*/g wet weight would carry ∼120 CFU/g dry weight. This is fourfold higher than the value reported in wet weight and significantly changes the interpretation of the result. The differences in methods chosen for analyzing poultry litter suggests that comparison of data across studies should be done carefully and cautiously.

Our intent is not to provide a validated method for further use by poultry researchers as the spiked litter experiment has several limitations: the absence of indigenous bacteria, low moisture, the weakly basic pH, and the age of the oven-dried litter. These properties are expected to affect the recovery of pathogens from litter and would require further studies involving many poultry research facilities and farms to determine their role in pathogen survival and recovery.

## Conclusion

Having accurate and efficient methods for pathogen quantification and detection from litter is one of the first steps to implementing pre-harvest reduction interventions. This study provides a template that poultry researchers can use when developing newer methods or for validation of current methods used for pathogen detection in litter. It is likely that method performance will differ by litter composition (e.g., pine shavings versus peanut hulls), litter age, and litter moisture; therefore, further studies to evaluate these differences are needed. Likewise, litter sampling methods such as drag/boot swabs require separate validation studies. In the short term, these studies will aid in the repeatability and reproducibility of litter studies and in the long-term assist in pathogen reduction in poultry pre-harvest.

## Data Availability Statement

The original contributions presented in the study are included in the article/Supplementary Material, further inquiries can be directed to the corresponding author.

## Author Contributions

JPL and AO designed the study. JPL and DC performed the experiment. JPL and AO wrote the manuscript. All authors revised and edited the manuscript.

## Author Disclaimer

Any opinions expressed in this article are those of the authors and do not necessarily reflect the official positions and policies of the USDA and any mention of products or trade names does not constitute recommendation for use. USDA is an Equal Opportunity Employer.

## Conflict of Interest

The authors declare that the research was conducted in the absence of any commercial or financial relationships that could be construed as a potential conflict of interest.

## Publisher’s Note

All claims expressed in this article are solely those of the authors and do not necessarily represent those of their affiliated organizations, or those of the publisher, the editors and the reviewers. Any product that may be evaluated in this article, or claim that may be made by its manufacturer, is not guaranteed or endorsed by the publisher.
