# Supplementary material for: Bacterial Detection and Recovery From Poultry Litter
Source: Front Microbiol. 2022 Jan 6;12:803150. doi: 10.3389/fmicb.2021.803150 (PMC8770916; doi:10.3389/fmicb.2021.803150)
Supplement: Supplementary file 2 [file Table_2.DOCX]

| Supplementary Table 2: Bacterial strains used for spiking poultry litter | | |
| --- | --- | --- |
| **Organism** | **Source** | **Antibiotic Resistance Profile** |
| *C. coli* | Poultry Litter | Tet |
| *C. coli* | Poultry Litter | Tet |
| *C. jejuni* | Poultry Litter | Pan Susceptible |
| *C. jejuni* | Poultry Litter | Pan Susceptible |
| *E. durans* | Poultry Litter | Lin Nit |
| *E. faecalis* | Poultry Litter | Erm Lin Tyl |
| *E. faecium* | Poultry Litter | Lin Nit |
| *E. hirae* | Poultry Litter | Lin |
| *E. coli* | Poultry Litter | Unk |
| *E. coli* | Poultry Litter | Unk |
| *E. coli* | Poultry Litter | Unk |
| *E. coli* | Poultry Litter | Unk |
| *S*. Enteritidis | Chicken Carcass Rinse | Pan Susceptible |
| *S*. Heidelberg | Chicken Carcass Rinse | Pan Susceptible |
| *S*. Kentucky | Chicken Carcass Rinse | Str |
| *S*. Typhimurium | Chicken Carcass Rinse | Amc Amp Tio Axo Fis Tet |
| Abbreviations: Erm (erythromycin); Lin (lincomycin); Nit (nitrofurantoin); Pan susceptible - susceptible to all antimicrobials tested; Tet (tetracycline); Tyl (tylosin); Unk (unknown); Str (streptomycin); Amc (amoxicillin/clavulanic acid); Amp (ampicillin), Tio (ceftiofur), Axo (ceftriaxone), Fis (sulfisoxazole) | | |

| Supplementary Table 3: Replicates positive by inoculum amount and detection method | | | | | | | | | | | |
| --- | --- | --- | --- | --- | --- | --- | --- | --- | --- | --- | --- |
| Inoculum Amount (cfu/g) | *Campylobacter* | |  | *E. coli* | |  | *Enterococcus* | |  | *Salmonella* | |
|  | direct plating^a^ | enrichment^a^ |  | direct plating^a^ | enrichment^a^ |  | direct plating^a^ | enrichment^a^ |  | direct plating^a^ | enrichment^a^ |
| 10^6^ | 2/3 | ND |  | 3/3 | ND |  | 3/3 | ND |  | 3/3 | ND |
| 10^5^ | 1/3 | ND |  | 3/3 | ND |  | 3/3 | ND |  | 3/3 | ND |
| 10^4^ | 3/3 | ND |  | 3/3 | ND |  | 3/3 | ND |  | 3/3 | ND |
| 10^3^ | 3/3 | ND |  | 3/3 | ND |  | 3/3 | ND |  | 3/3 | ND |
| 10^2^ | 0/3 | 2/3 |  | 3/3 | ND |  | 3/3 | ND |  | 3/3 | ND |
| 10^1^ | 0/3 | 0/3 |  | 1/3 | 3/3 |  | 0/3 | 3/3 |  | 2/3 | 3/3 |
| 10^0^ | 0/3 | 0/3 |  | 0/3 | 3/3 |  | 1/3 | 2/3 |  | 0/3 | 3/3 |
|  | | | | | | | | | | |  |
| ^a^ Plates with < 2 colonies were not included. | | | | | | | | | | | |
| ND, not determined. | | | | | | | | | | | |
