# Supplementary figures and images for: Bacterial Detection and Recovery From Poultry Litter
Source: Front Microbiol. 2022 Jan 6;12:803150. doi: 10.3389/fmicb.2021.803150 (PMC8770916; doi:10.3389/fmicb.2021.803150)

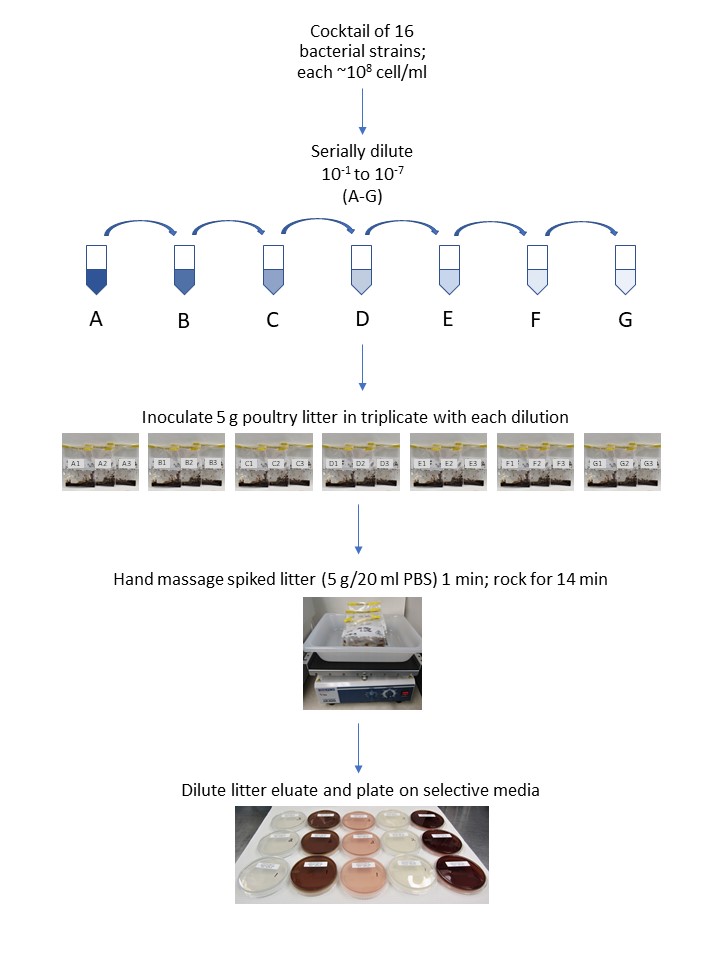


Supplementary Figure 1: Protocol used for litter inoculation study.

Supplement: Supplementary file 3 [file Table_3.DOCX]
